# Progress on Electrochemical Sensing of Pharmaceutical Drugs in Complex Biofluids

**DOI:** 10.3390/chemosensors11080467

**Published:** 2023-08-21

**Authors:** Elain Fu, Khadijeh Khederlou, Noël Lefevre, Stephen A. Ramsey, Matthew L. Johnston, Lael Wentland

**Affiliations:** 1 School of Chemical, Biological and Environmental Engineering, Oregon State University, Corvallis, OR 97331, USA; 2 School of Electrical Engineering and Computer Science, Oregon State University, Corvallis, OR 97331, USA

**Keywords:** pharmaceutical drugs, electrochemical detection, therapeutic drug monitoring, complex biofluids, companion diagnostic

## Abstract

Electrochemical detection, with its advantages of being rapid, multi-time point, compatible with cost-effective fabrication methods, and having the potential for miniaturization and portability, has great promise for point-of-care drug monitoring. However, a continuing challenge concerns the robust and sensitive electrochemical detection of pharmaceutical analytes from biological fluids. These complex matrices, such as saliva, sweat, interstitial fluid, urine, and blood/serum, contain multiple components that can contribute to an increased background or reduced analyte signal. In this mini-review, we discuss progress on electrochemical sensing in complex biofluids. We first introduce the challenge of drug titration in the management of various health conditions and provide an overview of the motivation for improved therapeutic drug monitoring, including current limitations. We then review progress on pharmaceutical drug detection from these biofluids with a focus on sample preprocessing, electrode modification for signal amplification, and/or electrode passivation to minimize fouling. Finally, we highlight promising strategies that have enabled robust drug quantification for clinical relevance and that may be useful for field-use systems.

## 1. Introduction

The electrochemical sensing of pharmaceuticals is an active area of research and development with multiple applications including environmental monitoring, pharmaceutical quality control, and diagnostics and therapeutic monitoring. Numerous previous reviews have described advances towards field-use systems for analyte monitoring. Sanavio and Krol (2015) described advances in the development of various point-of-care platforms as well as nanomaterials that could be used for therapeutic drug monitoring applications [1]. More recently, Sardini et al., (2020) reviewed printed electrochemical biosensors, i.e., sensors that use biological recognition elements, for a variety of target analytes including proteins, cells, and metabolites [2]. Zabihollahpoor et al., (2020) reviewed electrochemical sensors targeting antiseizure drugs for therapeutic drug monitoring [3]. In a complementary effort, Pollard et al., (2021) focused on electrochemical biosensors for therapeutic drug monitoring with an emphasis on promising sensing strategies that support continuous monitoring [4]. In a similar vein, Mobed et al., (2022) reviewed biosensors that specifically target antiseizure drugs [5]. Ozbek et al., (2022) reviewed potentiometric electrochemical sensors demonstrated using biological fluids and emphasized the limit of detection, recovery, and response time [6]. Kaur et al., (2022) [7] focused on electrochemical sensors using carbon-based nanomaterials for sensing drug and food pollutants. Most recently, Smith et al., (2023) [8] reviewed advances in the broad field of wearable sensing, while Calevilla et al., (2023) [9] reviewed work on sensors and biosensors applied to the monitoring of drugs for treating depression.

Here, we summarize recent progress (in the last five years) on the electrochemical sensing of pharmaceutical drugs in complex biofluids with an emphasis on addressing the challenge of detection in minimally processed samples. We first describe the motivation for the need for pharmaceutical monitoring by describing the challenge of drug titration in the management of many health conditions. Next, we provide an overview of the current practice of therapeutic drug monitoring, including specific drug classes of emphasis and describe the need for improved drug monitoring that would be enabled by field-use tools that can measure pharmaceuticals or their metabolic byproducts in complex biofluids. We then review progress in pharmaceutical drug detection from complex biofluids. We focus on sample preprocessing, electrode modification for signal amplification, and/or electrode passivation to minimize fouling and highlight the most promising strategies that would be useful for enabling personalized therapeutic drug monitoring in field-use systems. Finally, we close with a discussion of ongoing challenges in the achievement of point-of-care pharmaceutical monitoring systems.

## 2. Background

A major challenge in the management of health conditions that are treated using drug therapy is drug titration to control symptoms while minimizing adverse effects [10,11]. Drug titration can be further complicated in drugs with variable pharmacokinetics (PK) and strong interactions with other drugs. As an example, in the case of epilepsy, a neurological disease characterized by seizures that affects 65 million people worldwide [12], many of the antiseizure medications have serious toxicity risks, variable PK, and significant drug–drug interactions [13,14]. Thus, dose optimization can be difficult to achieve and may result in a reduced patient quality of life [15–17]. Drug overtreatment (dosing with higher than optimal drug levels in order to ensure seizure suppression) can further degrade patients’ quality of life, as well as lead to chronic health effects due to long-term drug use and/or medication noncompliance [18].

**Figure 1.**
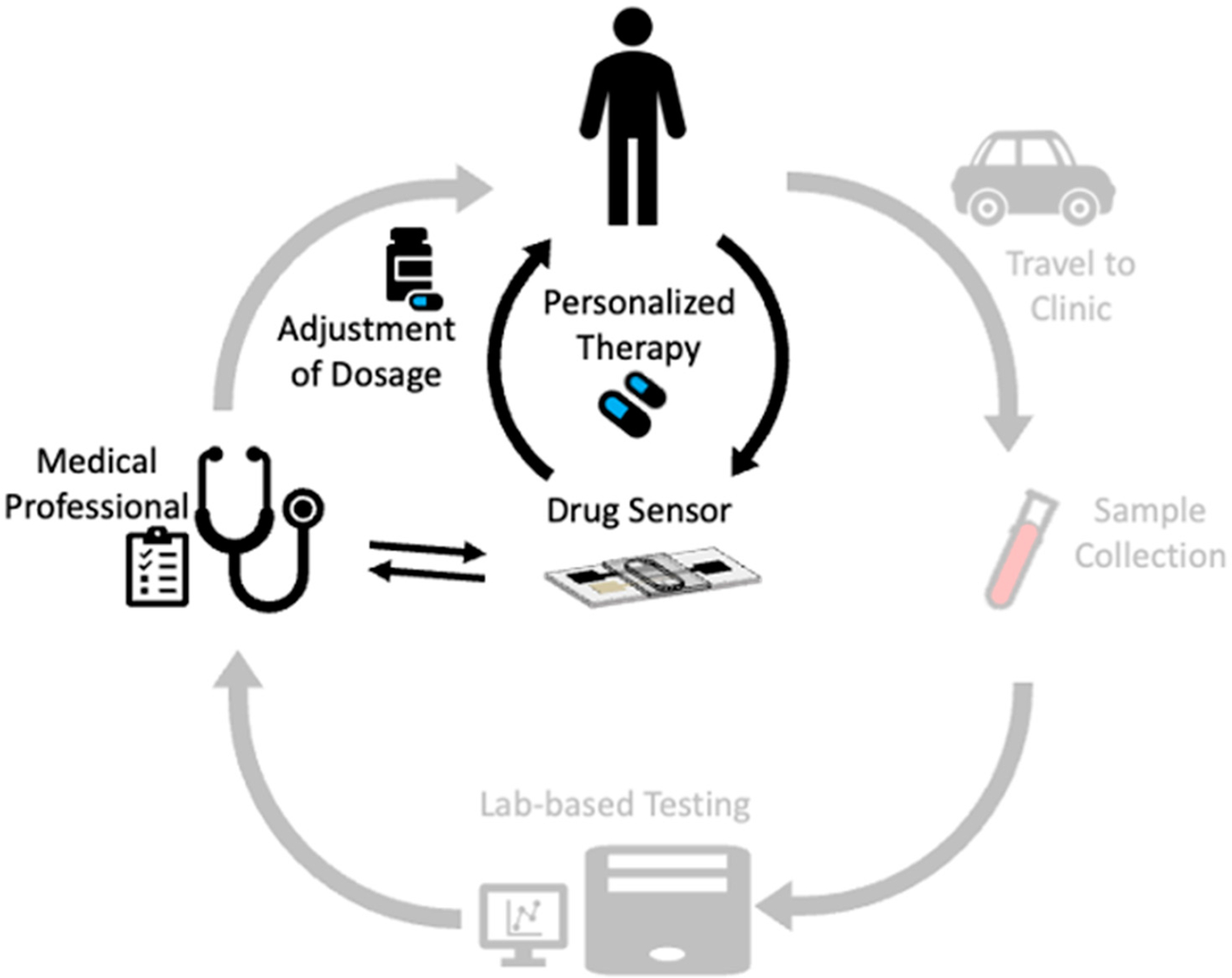
Traditional therapeutic drug monitoring requires clinic-based sample collection and lab-based analyses such that its utility is severely limited (outer cycle). The use of noninvasive biological fluids for sampling and field-use sensor systems for analysis could enable improved personalized therapeutic drug monitoring for each individual (inner cycle).

Therapeutic drug monitoring (TDM) has been discussed (and occasionally practiced) since the 1960s for multiple health conditions [10], including epilepsy [19]. It is based on the premise that drugs have a well-defined therapeutic range that can be broadly applied across the population. Although some clinicians currently use lab-based, blood-based TDM (Figure 1, outer cycle), the dependence on clinic sampling and lab analysis has limited its use to infrequent sampling. Further, the practice of TDM, which requires invasive venipuncture performed during a clinic visit and subsequent analyte quantification in a centralized lab, is a multi-day process that does not enable correlation of the patient drug level with their dosing schedule or experience with disease symptoms and adverse drug side effects. Prior work also indicates that substantial sex-based differences in PK can lead to adverse effects in women dosed using the established therapeutic ranges that were based on clinical trials composed mainly of men [20,21]. Likely as a result, the rate of adverse drug events has been reported to be much higher for women than men [20]. Finally, for each health condition, there are often specific subsets of patients who would benefit the most from multi-time point informed TDM. For example, women with epilepsy who are of childbearing age and, in particular, pregnant epilepsy patients, are high-risk populations for adverse drugs effects for themselves and the fetus [22–24].

In addition to therapeutic drug monitoring of antiseizure medication for epilepsy, there are numerous other classes of pharmaceuticals for which having a companion diagnostic device for drug monitoring could be beneficial. These include drugs used to treat other neurological conditions including Parkinson’s disease [25], drugs used in psychiatry [25] including those used to treat depression [26], drugs for the treatment of bacterial infections [27], and drugs for the treatment of viral infections such as hepatitis C [28]. Table 1 contains a summary of the classes of pharmaceuticals that are highlighted in this mini-review.

The use of noninvasive sampling fluids and point-of-care analysis could revolutionize TDM [48] and enable personalized TDM for each individual (Figure 1, inner cycle). Saliva [49,50], sweat [51], interstitial fluid [48], and urine [52], as alternatives to the gold-standard fluids of serum and blood, hold exceptional promise for point-of-care monitoring applications. For example, in the context of epilepsy management, the use of saliva as an alternative fluid for drug monitoring has been extensively discussed [53,54]. Critically, for monitoring applications in alternative biofluids, the drug concentration in the serum should be well-correlated with its concentration in the alternative biofluid. For example, in the context of antiseizure medication for epilepsy patients, the saliva concentration correlates strongly with the serum-free concentration at pharmacologically relevant levels [53,55–58] Given the straightforward, noninvasive nature of saliva, sweat, interstitial fluid, and urine collection, monitoring using these biofluids could enable multi-point, patient-controlled sampling to more comprehensively inform the patient’s individual therapeutic drug levels [1,53,54] and potentially improve healthcare.

We have chosen to organize this mini-review in sections based on the type of biofluid, since each biofluid has unique advantages and disadvantages related to its composition, variability across the population, volume, and accessibility. Table 2 contains a summary of the main components of each biofluid and their advantages and disadvantages.

## 3. Advances in the Electrochemical Detection of Pharmaceuticals in Blood-Based Biofluids

Advances have been made in electrochemical sensing in blood and serum/plasma, and these studies are summarized in Table 3. From the standpoint of pharmaceutical monitoring, serum has the advantage of being the gold-standard matrix for drug detection, and serum is considered to be relatively uniform across individuals. The disadvantages of blood-based biofluids are that the collection of moderate volumes requires venipuncture in a clinic. Further, processing serum/plasma from whole blood generally requires lab-based processing, including centrifugation. As an alternative to small-volume-requirement sensors, fingerstick blood volumes (20 μL) can be compatible with collection at the point of care, and field-use tools (e.g., commercially available membranes) exist for processing small volumes of whole blood to plasma.

Most of the studies reviewed on electrochemical sensing in blood-based biofluids relied on high levels of dilution (greater than five-fold and sometimes upwards of 100-fold) or separation of the serum protein content before the electrochemical analysis. Only one of the studies, which focused on a field-use sensor, demonstrated detection from a finger-stick blood sample that would be compatible for sampling at the point of care [61]. However, the sensor still required dilution of the whole blood sample by 20-fold before analysis [61]. Thus, the ability to detect the target drug analyte directly in a complex matrix was not emphasized in the set of blood-based studies reviewed.

Progress in the electrochemical sensing of drug targets in blood-based biofluids has been made on other fronts, including signal enhancements using novel and more traditional electrode modifications, as well as the simultaneous monitoring of multiple target analytes (e.g., multiple drugs and/or potential interferents [62]). Given our focus on electrochemical monitoring to support drug titration, for each of the studies reviewed in Table 3, we highlight the drug target(s), the electrochemical method with the base electrode and sensing mechanism, the complex biofluid and any associated processing performed, the main cited strategies to improve the electrochemical signal, and the detection performance (e.g., limit of detection, dynamic range, and recoveries). Most of the sensors reviewed in this section used conventional carbon-based working electrodes composed of glassy carbon or carbon paste and then subsequently modified with nanostructures to enhance the electrochemical signal. Strategies for signal enhancement included the use of carbon-based materials such as multi-walled carbon nanotubes (well-known for having favorable properties such as a large surface area, high electrical conductivity, and resistance to fouling [63]) and graphene (also known for its large surface area, electrical conductivity, and mechanical strength [63]) in combination with other nanostructures. The nanostructures used varied from single metal nanoparticles composed of gold or cobalt to bi-metallic combinations of platinum-nickel and platinum with palladium to metal oxide nanoparticles such as zinc oxide and zirconium oxide. Further, more complex, novel combinations of layered structures were used for signal enhancement that included ionic liquid crystals and molecularly imprinted polymers. These modifications were utilized to generate robust electrochemical signals acquired using a variety of electrochemical methods, but most often via differential pulse voltammetry (DPV) or square-wave voltammetry (SWV) for drug target quantification.

**Figure 2.**
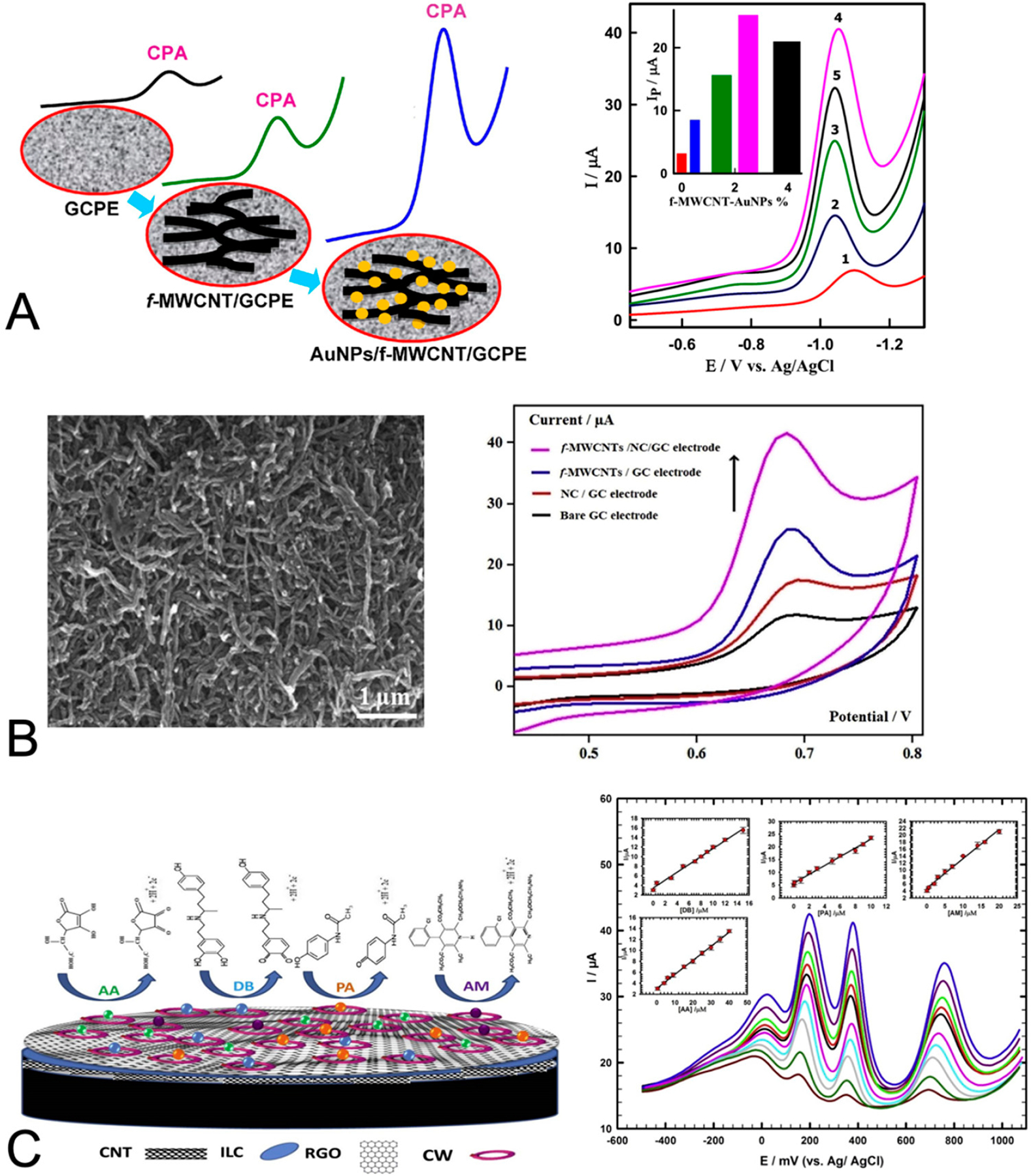
Strategies for signal-enhanced drug monitoring using modifications to the base carbon electrodes. (**A**) Ibrahim et al. [64] demonstrated the quantitative detection of cyproterone acetate using glassy carbon electrodes modified with multi-walled carbon nanotubes and gold nanoparticles (**left**). This strategy resulted in a substantial increase in the current peak of the square-wave voltammogram relative to the case of the base electrode only (**right**). The voltammograms represent cyproterone acetate (4.2 μM) acquired using electrodes containing 0% (1), 0.5% (2), 1.5% (3), 2.5% (4), and 4% (5) of their nanocomposite modification (as shown in the inset plot). Reproduced with permission from Elsevier. (**B**) Shalauddin et al. [65] used a combination of nanocellulose and multi-walled carbon nanotubes (SEM image, **left**) to modify their base glassy carbon electrodes and demonstrated an improved and enhanced signal of diclofenac sodium using cyclic voltammetry (**right**). Reproduced with permission from Elsevier. (**C**) Atta et al. [62] used a composite of carbon nanotubes, ionic liquid crystals, reduced graphene oxide, and 18-Crown-6 to modify their glassy carbon electrode (**left**). They demonstrated the simultaneous detection of amlodipine, acetaminophen, dobutamine, and ascorbic acid using differential pulse voltammetry (**right**, analyte peaks from right to left); the voltammograms represent increasing concentrations of analytes in the ranges, 0.02 to 20 μM, 0.004 to 10 μM, 0.02 to 15 μM, and 0.4 to 40 μM, respectively. Reproduced with permission from Elsevier.

Examples of notable signal enhancement strategies used in blood-based diagnostics are highlighted in Figure 2. Ibrahim et al. [64] demonstrated the quantitative detection of cyproterone acetate, an anti-androgen drug used in cancer treatment. Their sensor used a base electrode composed of glassy carbon paste that was then modified with a composite of multi-walled carbon nanotubes and gold nanoparticles. They optimized the concentrations of gold nanoparticles and multi-walled carbon nanotubes to achieve a substantial increase in the signal over the untreated electrode case (Figure 2A). Shalauddin et al. [65] targeted diclofenac sodium, an analgesic and anti-inflammatory drug used to treat arthritis and other conditions. In their sensor, they modified a base glassy carbon electrode with a composite of nanocellulose and multi-walled carbon nanotubes. They demonstrated significant gains in the signal using a combination of nanocellulose and multi-walled carbon nanotubes compared to using each component alone (Figure 2B). Atta et al. [62] demonstrated the simultaneous detection of multiple small molecule analytes, dobutamine, and amlodipine for the treatment of cardiac conditions, as well as acetaminophen and ascorbic acid. They modified a base glassy carbon electrode with a composite of multi-walled carbon nanotubes, ionic liquid crystals, graphene, and 18-Crown-6. Using differential pulse voltammetry, they showed well-resolved detection of the four different small molecule targets (Figure 2C).

**Figure 3.**
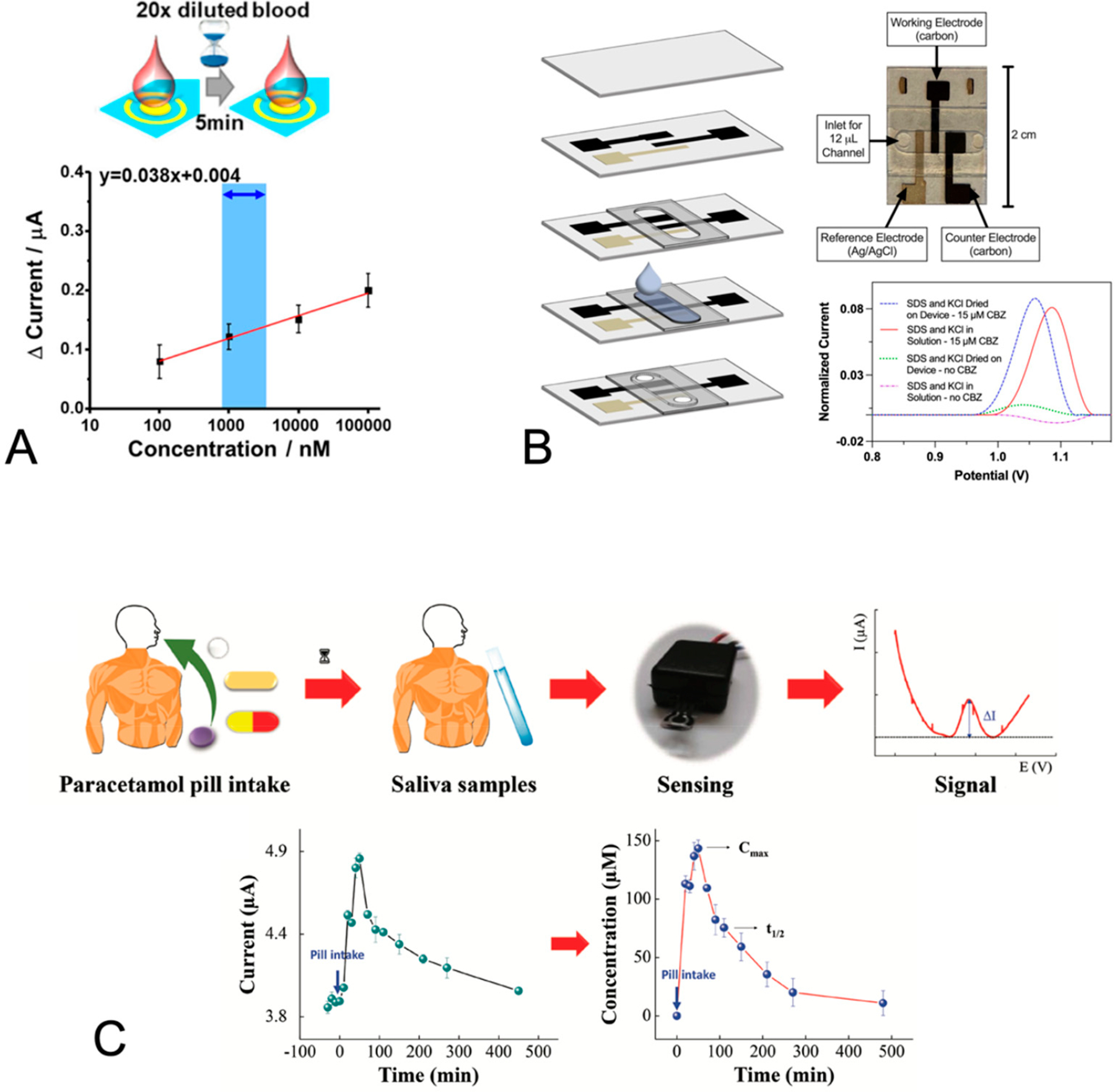
Advances in monitoring at the point of care. (**A**) Chung et al. [61] demonstrated the sensing of carbamazepine using a (diluted) fingerstick blood sample (**top**). In their electrochemical sensor, aptamer binding to carbamazepine resulted in an increased current signal from colocalization of the methylene blue tag with the electrode and enabled carbamazepine quantification down to 10 nM (**bottom**). Reproduced from [61]. (**B**) Wentland et al. [79,80] used the anionic surfactant sodium dodecyl sulfate (SDS) to facilitate the electrochemical detection of carbamazepine against a background of commercially purchased saliva. Their field-use compatible flow cell that included a dry SDS film on stencil-printed electrodes (**left** and **right top**) produced a similarly enhanced signal to that produced from SDS in solution (**right bottom**). Reproduced from [79,80]. (**C**) Gomes et al. [81] demonstrated acetaminophen monitoring from the unprocessed saliva of a healthy subject (**top**). Their sensor, pretreated using cyclic voltammetry of a sulfuric acid solution, enabled the tracking of the drug concentration over time (**bottom**). Reproduced from [81].

Complementary to the work performed using carbon-based electrodes, Chung et al. [61] described the development of an aptamer-based sensor for carbamazepine, an antiseizure drug that is widely prescribed for epilepsy. Their sensor used gold electrodes onto which an aptamer specific to carbamazepine was attached via a thiol [61]. The aptamer was tagged with methylene blue, such that upon the binding of the aptamer to the drug target, the methylene blue was localized close to the electrode and increased the electron transfer rate and current signal [61]. A key feature of their sensor was the high packing density of the aptamer to support sensitive drug detection. The authors demonstrated the assay selectivity against interference by analogues of carbamazepine and carbamazepine quantification within 5 min in diluted fingerstick blood (see Figure 3A).

## 4. Advances in the Electrochemical Detection of Pharmaceuticals in Alternative Fluids

Advances have also been made in electrochemical sensing in alternative biofluids to blood/serum/plasma, and these studies are summarized in Table 4. In the following discussion, we further highlight the strategies described that have supported robust drug quantification in alternative biofluids with an emphasis on methods that address unique challenges presented by complex biofluids, including an enhanced electrochemical signal against a high background, distinguishing the target signal in the presence of specific interference, and the prevention of electrode fouling.

### 4.1. Detection of Analyte Drugs in Saliva

Saliva has long been considered a promising biofluid for analyte detection due to its ease of collection, i.e., a pain-free, rapid process that can be performed ‘on demand’. However, saliva poses multiple challenges that have hindered its broader utilization in TDM. Saliva has a complex composition that varies across individuals in the population, as well as within an individual from day-to-day. Saliva is mainly composed of water and ions that give it a buffering capacity [59]; ion concentrations can change based on the saliva flow rate, e.g., whether the saliva is stimulated or unstimulated [59]. Saliva contains many different proteins, and their concentrations may vary based on the flow rate and physiological state of the cardiovascular system [59]. Saliva also contains hormones as well as small concentrations of uric acid, glucose, amino acids, lipids, and fatty acids [59]. Due to this complex composition of saliva and the associated potential for signal interference, the quantification of analytes in saliva can be challenging. Further, the analyte concentration in saliva is often at substantially lower levels than in the serum. Thus, strategies to enhance the target analyte signal over that of the background and interferents are critical for robust analyte quantification in saliva.

Several groups have reported different strategies to address the high background caused by interferents in saliva. Wentland et al. investigated a series of signal enhancement strategies to enable the detection of the antiseizure medication carbamazepine in commercially purchased pooled saliva [79]. They demonstrated that the use of the anionic surfactant sodium dodecyl sulfate (SDS) (as a facilitator of the interaction of carbamazepine with the electrode) combined with incubation resulted in a substantially increased carbamazepine signal compared to that with the bare stencil-printed carbon electrode [79]. In a follow up study, they demonstrated progress towards a field-use system composed of a disposable electrochemical flow cell (with all reagents incorporated dry) and a miniature potentiostat that enabled carbamazepine quantification at therapeutically relevant levels in saliva (see Figure 3B) [80]. Additional work is still needed to address the inter- and intra-individual variability of saliva.

**Figure 4.**
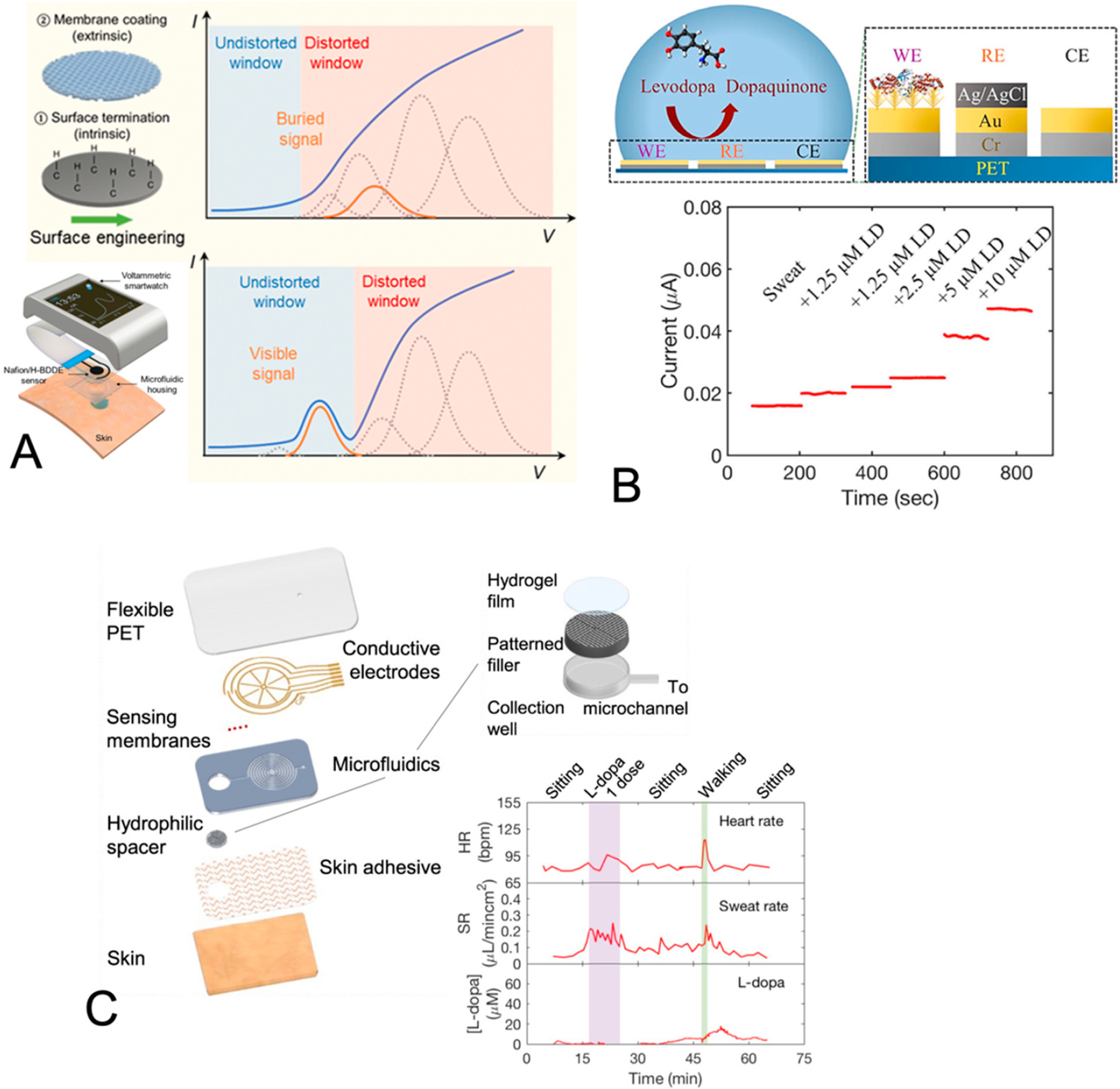
Advances in the electrochemical monitoring of drug analytes in sweat. (**A**) Lin et al. [82] developed a sensor for acetaminophen in sweat (**left**). A highlight of their design was the surface engineering of their boron-doped diamond electrode with a passivation layer combined with a Nafion coating to increase the peak current as well as to shift the peak potential for acetaminophen away from the potentials of interferents (**right**). In the schematic plot, orange corresponds to the analyte of interest, dashed grey to interferents, and blue to the overall signal. Reproduced from [82]. (**B**) Tai et al. [83] reported the development of an arm band sensor for levodopa monitoring in stimulated sweat. Their sensor design uses gold electrodes on a plastic substrate to measure the tyrosinase-catalyzed oxidation of levodopa (**top**). The sensor showed an increasing current signal for greater concentrations of levodopa against a background of sweat (**bottom**). Reprinted with permission from [83]. Copyright 2019 American Chemical Society. (**C**) Nyein et al. [84] developed a wearable patch that enables the monitoring of levodopa in sweat at rest. Their multilayer design also includes a hydrogel layer that is coupled to a hydrophilic spacer for the transport of small volumes of sweat from the skin to the downstream microfluidic channel for analysis (**left**). Their patch can simultaneously measure heart rate, sweat rate, and sweat levodopa levels (also via tyrosinase-catalyzed oxidation) in situ as demonstrated in a healthy human subject after consuming L-dopa containing broad beans (**right**). Reproduced from [84].

In a complementary effort, Lin et al. [82] demonstrated the use of targeted surface engineering to create a ‘nondistorted potential window’ for the detection of the analyte acetaminophen in saliva with the interferents tyrosine, tryptophan, and uric acid. They accomplished this through a combination of hydrogen passivation of the base boron-doped diamond electrode and a nonfouling Nafion layer that both enhanced and shifted the acetaminophen signal [82]. The investigators noted the use of saliva centrifugation before analysis (i.e., the removal of cellular debris and higher-molecular-weight proteins from the saliva matrix), so the sensor robustness against unprocessed saliva was not determined [82]. The sensor was also demonstrated against a background of unprocessed sweat (see Figure 4A) [82].

Most recently, Gomes and Raymundo-Pereira demonstrated the detection of acetaminophen against a background of undiluted saliva using differential pulse voltammetry and screen-printed carbon electrodes (see Figure 3C) [81]. Notably, the authors showed the detection of their drug target against a background of undiluted saliva collected via the passive drool method [81]. Further they showed time course data of the drug level in saliva before and after taking a drug dose [81]. The authors attributed the high sensitivity of their sensor and its robustness to fouling to their electrode pretreatment with sulfuric acid [81] but did not quantify the difference in performance. A discussion of possible strategies for the implementation of the pretreatment step in a point-of-care device would have been useful.

### 4.2. Detection of Analyte Drugs in Sweat

Sweat is another biofluid for which there has been much interest due to its potential for noninvasive continuous sampling. Sweat is made up of 99% water, and the main components of the other 1% are electrolytes, fatty acids, lactic acid, and multiple nitrogen metabolites, including uric acid, urea, and ammonia [51]. Sweat also contains small amounts of proteins (including antibodies), peptides, amino acids, amines, metal ions, and ethanol [51]. De Castro et al. [51] have argued that sweat has high impact potential as a biofluid for use in drug sensing. The challenges of using sweat as a monitoring fluid include its low resting secretion rates (10 to 100 nL/min per cm^2^) and the need to minimize evaporation effects in order to obtain an adequate biofluid volume for accurate analysis [34].

Several groups have reported advances in the electrochemical detection of pharmaceuticals in sweat. Levodopa, a drug used in the treatment of Parkinson’s disease, has been a common target due to its short half-life and the fact that optimal dosing becomes more difficult as the disease progresses. The Javey group described the development of an on-the-arm wearable device for monitoring levodopa using amperometry (see Figure 4B) [83]. The device electrodes were composed of gold nanodendrites deposited on a thin gold/chromium layer and then modified by deposition of thionin acetate salts [83]. Further modifications to the working electrode included a layer of glutaraldehyde-crosslinked tyrosinase and then Nafion [83]. Levodopa quantification was achieved via enzyme-facilitated levodopa oxidation. The authors noted the key features of their sensor as being the use of gold nanodentrites to increase the surface area for enzyme attachment and the use of Nafion film as a nonfouling layer that provides sensor signal stability in the longer-term [83]. They demonstrated levodopa quantification of their sensor in the range of 0 to 20 μM against a background of sweat, as well as the monitoring capability of their sensor on human subjects who had ingested levodopa-containing fava beans and using simulated sweat via iontophoretic stimulation or exercise [83].

In a follow-up study, the Javey group further advanced sweat sensing by demonstrating a system to monitor levodopa in subjects at rest (see Figure 4C) [84]. A key feature of their sensor system that was critical in facilitating the collection of the very small volumes of sweat produced during the resting state was the human interface component composed of an agarose-glycerol hydrogel film on a PVA-coated SU-8 [84]. The sensor also included a Nafion coating to resist fouling [84]. Further, in order to provide accurate quantification of levodopa levels over time, their system included impedance-based sweat secretion rate monitoring using a configuration of interdigitated electrodes [84]. And, the miniature footprint of the system was also key to the achievement of conformal placement on the target areas of the fingertip and wrist while allowing for the performance of daily activities [84]. Against a background of sweat, the levodopa sensor was characterized as having improved sensitivity over their prior work, despite a decrease in the sensing area [84]. Finally, the investigators demonstrated on-the-body sweat monitoring that showed increasing levodopa levels with increasing doses of broad bean consumption [84].

The Wang group also reported the development of their touch-based levodopa sensor for sweat sensing [85]. Their sensor uses screen-printed carbon electrodes modified with crosslinked tyrosinase and amperometry [85]. Similar to the studies described above, the electrochemical sensor utilizes the tyrosinase-facilitated oxidation of levodopa. However, in this sensor design, the levodopa concentration is tracked via dopaquinone reduction after its conversion from levodopa [85]. The investigators noted that an advantage to their method is its robustness to fouling of the electrode via unintended quinone polymerization reactions [85]. Notably, simultaneous measurements of levodopa levels in capillary blood showed a correlation with the levodopa levels in nonstimulated sweat [85].

More recently, Raymundo-Pereira et al. [86] reported the development of a wearable sensor in glove format for monitoring the pharmaceuticals acetaminophen and paroxetine. The sensor was characterized for its mechanical stability, selectivity, and response in artificial sweat, as well as the reproducibility of a single drug concentration against a background of human sweat combined with artificial sweat [86]. The investigators noted the requirement of electrode pretreatment with an acidic solution in order to reduce impurities and achieve the desired sensor response [86].

### 4.3. Detection of Analyte Drugs in Interstitial Fluid

Interstitial fluid (ISF) is another complex matrix for which there is much interest due to its potential for noninvasive continuous sampling. The main components of ISF are amino acids, carbohydrates, and fatty acids [48]. Further, drugs and small micronutrients are able to pass through the capillary walls into the ISF [48]. Aside from the fact that it can be noninvasively sampled, ISF is preferred over blood samples because it does not contain proteins and other cells that can interfere with drug-level analysis [48], and thus, it does not require the extensive sample manipulations required to process whole blood to serum.

The two main challenges associated with sensing in ISF are the very small fluid volumes that must be accessed from the extracellular spaces and then directed to a microelectrode system for detection and the resistance to biofouling of the electrodes over the timescale of monitoring with the microelectrodes. The Wang group reported on their promising microneedle-based electrochemical sensing platform in which carbon paste electrodes and a silver wire reference electrode are embedded into hollow microneedles [87]. Goud et al., applied their platform to the detection of levodopa by combining tyrosinase with the carbon paste in order to monitor the enzyme-facilitated oxidation of levodopa using amperometry [88]. In addition, a Nafion coating was included to provide resistance to interference by negative species [88] and to reduce electrode fouling by larger-molecular-weight proteins [63].

**Figure 5.**
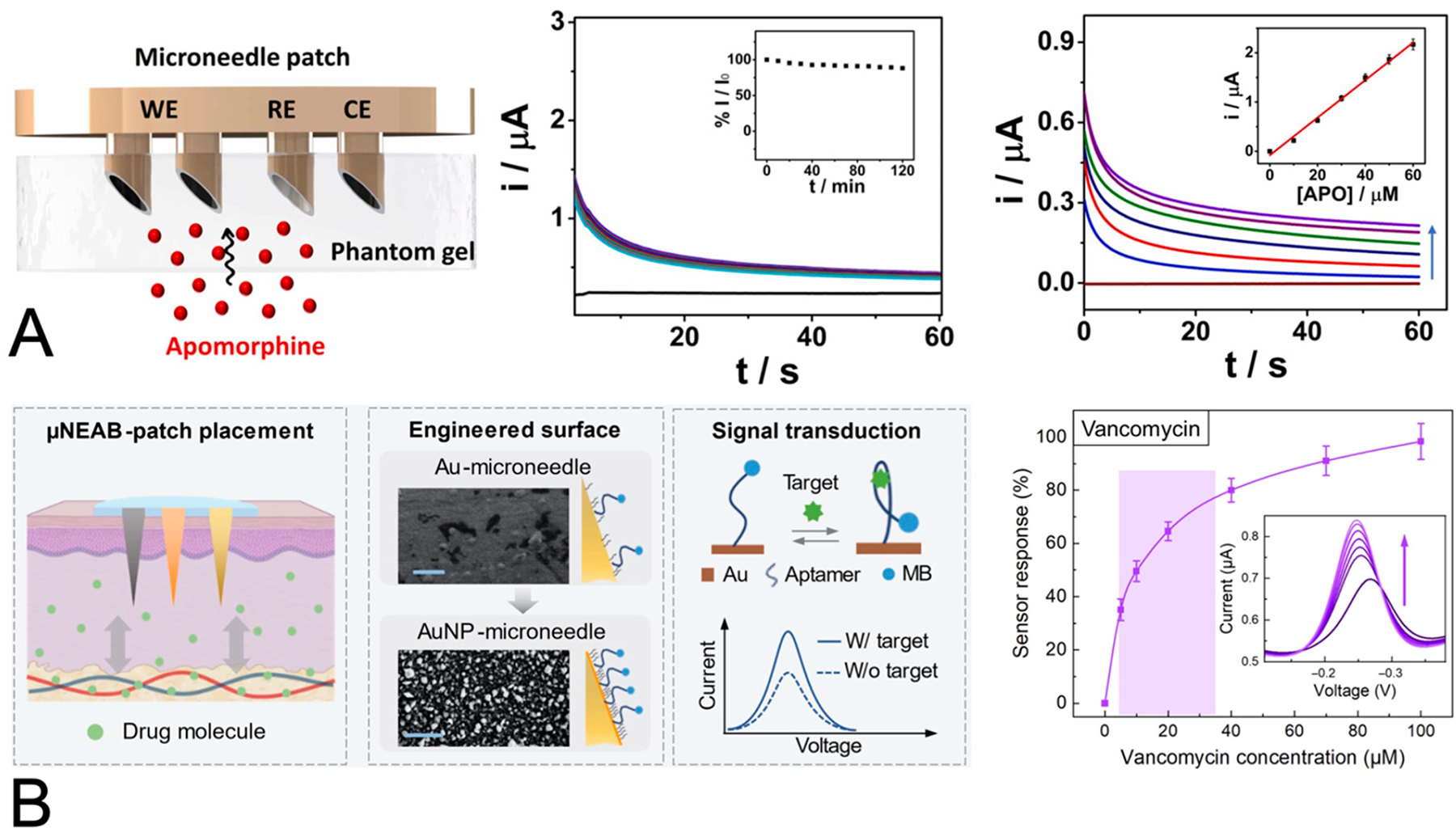
Advances in electrode integration into microneedles for the electrochemical sensing of drug analytes in ISF. (**A**) Goud et al. [89] developed an electrochemical sensor for the monitoring of apomorphine in ISF. A highlight of their sensor design is the integration of modified carbon paste electrodes into 3D printed microneedles. The electrodes were coated with a 1% Nafion film to minimize fouling from protein interferents. The addition of 2% rhodium nanoparticles was demonstrated to increase the signal generated for the oxidation of apomorphine. Their sensor showed stability across multiple measurements (10 with intervals of 10 min) and quantitative response across concentrations from 0 to 60 μM. Reproduced from [89] with permission from Elsevier. (**B**) Lin et al. [90] developed an electrochemical sensor for monitoring antibiotics in ISF. Their design uses electrodes composed of gold-coated acupuncture needles. The sensing mechanism used aptamers tagged with methylene blue, such that aptameter binding to the antibiotic results in the positioning of the methylene blue in closer proximity to the electrode and an increase in the current signal from the methylene blue activity. Their sensor showed quantitative response across vancomycin concentrations from 0 to 100 μM (arrow denotes increasing concentrations for the series of voltammograms). Reproduced from [90].

In a follow up study, the Wang group demonstrated the detection of apomorphine, another drug used in the treatment of Parkinson’s disease [89]. The investigators demonstrated that doping the carbon paste working electrode with rhodium nanoparticles resulted in larger differences in the current peaks compared to the situation without doping and thus increased the resolution of the apomorphine concentrations of interest using artificial ISF within a gel as a test bed [89]. A nonfouling layer of Nafion was incorporated on the working electrode, and with it, the authors reported the successful detection of apomorphine over potential interferents including histidine, phenylamine, tryptophan, and acetaminophen [89] (see Figure 5A).

In a complementary effort, the Emaminejad group [90] demonstrated a microneedle-based electrochemical sensor for two antibiotics, tobramycin and vancomycin, with narrow therapeutic ranges and for which therapeutic drug monitoring has been indicated. Their wearable sensor uses gold-nanoparticle-coated acupuncture needles fixed within a PDMS substrate [90]. Aptamers, functionalized with the redox reporter methylene blue, were attached to the electrode surface, such that their conformational change during target binding resulted in a detectable change in the voltammetric signal [90]. The authors noted that their system was robust to biofouling, as demonstrated by the limited drift when exposed to a protein-spiked buffer over 14 h [90]. The highlights of the sensor’s capabilities include in vivo (rat) correlation studies of the drug concentration in serum vs. ISF [90] (see Figure 5B).

### 4.4. Detection of Analyte Drugs in Urine

Urine is a complex biological fluid, comprising water, urea, uric acid, inorganic salts, enzymes, nucleic acids, vitamins, proteins, amino acids, hormones, mesothelin, beta-microglobuin, urokinase, antibiotics, and mycomycin [52]. Urine collection has the advantage of being noninvasive and easy to sample, and there may be a longer time window available for drug detection compared to other biological matrices such as saliva or blood [60]. However, urine samples cannot be collected ‘on demand’ and may be contaminated if the collection is not conducted carefully [60].

As was the case for the studies reviewed on blood-based biofluids, the majority of studies reporting on electrochemical drug sensing in urine have relied heavily on processing to remove the protein content or sample dilution in order to mitigate interference effects. Only one study by Ishii et al. demonstrated drug quantification in unprocessed urine [91]. In that work, the investigators used boron-doped diamond (BDD) electrodes to monitor the reduction of the diuretic triamterene. The rationale for the choice of the BDD electrode include its resistance to biofouling, its stability, and its relatively large potential window [91]. The choice to monitor the triamterene reduction was motivated by the occurrence of high background current at higher potentials. When assessing the drug content in individual urine samples, high levels of ascorbic acid were found to be problematic, as they interfered with the triamterene signal [91]. Also noteworthy was the variability of the triamterene potential in the different individual urine samples and the potential need for more sophisticated signal analysis algorithms.

Notable signal enhancement strategies used in studies on urine-based electrochemical sensing overlap with the studies highlighted using blood-based biofluids and include the use of multi-walled carbon nanotubes combined with gold nanoparticles [64], palladium and platinum nanoparticles [70], or nanocellulose [65]. Other reported electrode modifications for enhanced electrochemical signals include platinum nanoflowers with reduced graphene oxide [92] and a composite of iron oxide and polypyrrole and palladium [93]. In addition, multiple reports describe the use of molecularly imprinted polymers [73,94–96].

## 5. Summary and Ongoing Challenges

There has been exciting progress in the development of electrochemical sensing methods in a variety of biofluids. The studies reviewed investigated numerous drugs with strong clinical motivation for monitoring and have reported effective strategies for signal enhancement and mitigating the effects of interferents. In addition to electrode design to increase the conductivity and electroactive surface area, a key requirement for sensing in biofluids is the judicious choice of electrode materials/modifications to increase the fouling resistance and thus improve the signal stability. In the studies reviewed, choices for electrode modification often included multi-walled carbon nanotubes and graphene, materials that have been reported to have fouling resistance to particular species of interest [63]. Further, multiple studies have incorporated surfactant or Nafion coatings to mitigate electrode fouling by negatively charged species and/or larger-molecular weight-species such as proteins.

An unaddressed challenge in this area of electrochemical detection of pharmaceuticals is achieving robust analyte quantification from biofluids at the point of care. Most of the studies that performed measurements in blood and urine that were reviewed here relied on high levels of sample dilution (it is important to note that point-of-care detection was not necessarily a priority for all studies; some may have assumed the current status quo for patient sample collection in a clinic and subsequent lab-based analysis). Sample dilution could be implemented at the point of care, but would require either (i) additional user steps to manually perform the dilution or (ii) additional complexity (and cost) of the device in the form of an upstream sample processing module that automatically performs the dilution ([101] describes a microfluidic implementation demonstrated for blood processing and [102] describes H-filter enrichment of a drug over larger-molecular-weight interferents in the context of saliva processing). Further, sample dilution of the analyte would require a higher sensitivity point-of-care sensor, thus entailing the classic challenge of achieving high sensitivity in a field-use format.

For the studies that performed measurements in unprocessed biofluids, multiple challenges remain. Biofluid variability can be significant over time (e.g., daily for saliva) in an individual, as well as across the population. Thus, there is a need for more studies to assess electrochemical methods in raw samples without substantial processing as well as to validate methods using biofluid samples from many different individuals to assess the robustness more comprehensively.

Finally, the realization of effective companion diagnostics will require the thoughtful use of fabrication materials and methods that are compatible with field-use formats and that meet the requirements of usability, cost, maintenance, and performance. For example, with regard to fabrication, multiple articles cited in this review used either stencil- or screen-printed electrodes, methods which are consistent with a low cost per device and the ability to scale up. Additionally, progress has been reported in low-cost fabrication methods, including the use of stencil-printing materials other than carbon ink (e.g., glassy carbon [103]) and the use of the screen-printing process for modifications such as Nafion^™^ [104]. These advances could be promising for incorporation in future work targeting electrochemical sensing for pharmaceutical detection at the point of care.

## Figures and Tables

**Table 1.** Classes of pharmaceuticals for which drug monitoring could be beneficial.

Class	Example(s) from the Studies Reviewed	Motivation for Drug Monitoring
Drugs to suppress seizures in the treatment of epilepsy	Carbamazepine	Highly variable pharmacokinetics; strong interactions with other common drugs; and/or high toxicity [13,14]
Drugs to treat bacterial or fungal infections	Tobramycin; vancomycin; levofloxacin; rifampicin; azithromycin; sulfanilamide; trimethoprim; ketoconazole	Potential for kidney injury for tobramycin [29]
Drugs to treat Parkinson’s disease	Apomorphine; levodopa	Side effects of nausea for apomorphine [30] and decreased efficacy and increased motor disturbances with use for levodopa [31]
Drugs to treat depression	Paroxetine; benzodiazepines	Highly variable pharmacokinetics with a longer time for clearance with aging and renal/hepatic damage for paroxetine [26]
Drugs to treat cancer	Methotrexate; doxorubicin; dasatinib; epirubicin; cyproterone acetate; regorafenib; interferon gamma	Pulmonary and hepatotoxicity for methotrexate [32]; cardiotoxicity for doxorubicin [33]; adverse effects include dyspnea, fatigue, nausea for dasatinib [34]; hepatotoxicity for epirubicin [35]; hepatotoxicity for cyproterone acetate [36]; adverse effects include dyspnea, fatigue, nausea for regorafenib [37]
Drugs to treat hepatitis C viral infection	Daclatasvir; sofosbuvir; ledipasvir	Potential adverse drug–drug interactions for transplant and HIV patients [38]
Drugs to treat psychiatric disorders	Olanzapine	Side effects of overdose such as nausea, slurred speech, vomiting, damage to the aorta resulting in bleeding or death [39]
Drugs to treat cardiac conditions	Etilefrine; epinephrine	Overdosing on etilefrine can cause heart failure, hypertension, and erectile dysfunction [40]; epinephrine has interactions with other common compounds [41]
Anti-inflammatory and analgesic	Mefenamic acid; diclofenac sodium	Potential for renal toxicity [42,43]
Analgesic	Acetaminophen/paracetamol; nalbuphine	Hepatotoxicity [44] and nephrotoxicity [45] for acetaminophen; potential for TDM in neonates for nalbuphine [46]
Drugs for the treatment of bronchial asthma	Aminophylline	Potential for drug-induced cardiotoxicity [47]

**Table 2.** Summary of the composition and challenges of drug analyte detection in complex matrices.

Complex Biofluid	Major Components	Advantages	Disadvantages
Blood	Ions, proteins, glucose, amino acids, lipids, hormones, erythrocytes, leukocytes, platelets [49]	Gold standard; uniform across individuals; small fingerstick volumes (20 μL) are compatible with point-of-care collection	Invasive and painful; larger venipuncture volumes require a phlebotomist, which is inconvenient and limited to low-frequency collection
Serum	Ions, proteins, glucose, amino acids, lipids, hormones [49]		
Saliva	Ions, small molecules, proteins, mucins, hormones, blood-derived compounds, food debris, uric acid [59]	Noninvasive; moderate (1 mL) volume; easy to sample; frequent donation possible and on demand; could be compatible with continuous wearable device	Properties variable across individuals; variability throughout the day for individuals including pH; possible food contamination
Sweat	Ions, small molecules, proteins, pyruvate, lactate urea, antigens, antibodies, ethanol [51]	Noninvasive; compatible with continuous wearable devices	Low secretion rate (10 to 100 nL/min per cm^2^) volume unless stimulated; variability in the rate secreted; possible contamination from cosmetics or the environment
Urine	Inorganic salts, urea, uric acid, proteins, enzymes, nucleic acids, vitamins, hormones, amino acids, mesothelin, beta-microglobulin, antibiotics, urokinase, mycomycin [52]	Noninvasive; large (many mL) volume; easy to sample; there may be a longer time window available for drug detection compared to other biological matrices such as saliva or blood [60]	Sampling is not always possible ‘on demand’; contamination potential if the collection is not conducted carefully [60]
Interstitial fluid	Amino acids, carbohydrates, fatty acids [48]	Noninvasive; compatible with continuous wearable devices	Very small (nL) volume unless suction used

**Table 3.** Summary of advances in drug detection in blood and serum.

Drug and Health Condition	Electrochemical Method/Base Working Electrode/Sensing Mechanism	Complex Biofluid	Strategies to Improve Electrochemical Signal	Performance Metrics (Note that Metrics are Provided for Each of the Targets in the Order Listed in Column 1)	Ref.
Carbamazepine to treat epilepsy seizures	SWV; gold electrode; aptamer binding to carbamazepine reduces the distance between the methylene blue tag and the electrode and increases the current signal	Fingerstick blood-diluted 20-fold	High packing density of aptamer for increased sensitivity to the target	LOD 2.1 nM in serum for 5 min and linearly over the 17 to 51 μM therapeutic range	[61]
Daclatasvir, sofosbuvir, and ledipasvir for the treatment of hepatitis C	DPV; glassy carbon electrodes; oxidation of each of the drugs	Serum-diluted 200-fold	Multi-walled carbon nanotubes in an ionic liquid crystal and cobalt nanoparticles	LODs 1.9 nM, 7.3 nM, 0.28 nM; linear DRs of 0.07 to 1 μM and 5 to 50 μM, 0.3 to 8 μM and 10 to 100 μM, 0.02 to 1 μM and 3 to 100 μM; recoveries of 99.7 to 102.8% for ledipasvir	[66]
Cyproterone acetate for the treatment of prostate cancer	SWV; glassy carbon paste electrodes; reduction of cyproterone acetate	Serum-proteins separated out with ethanol precipitation and centrifugation	Multi-walled carbon nanotubes and gold nanoparticles	LOD 17.7 nM; linear DR of 99 nM to 8.3 μM; sensitivity 117 μA/μM per cm^2^	[64]
Regorafenib for the treatment of hepatocellular carcinoma	DPV; glassy carbon electrodes; oxidation of regorafenib	Serum-diluted 50-fold	Zirconium oxide nanoparticles and reduced graphene oxide	LOD 17 nM in buffer (not reported in serum); linear DR of 11 to 343 nM in buffer; recoveries of 97.2 to 102.6%	[40]
Doxorubicin and dasatinib for treatment of breast cancer	CA and SWV; carbon paste electrodes; oxidation of drugs	Serum-handling was not described	Zinc oxide nanoparticles and butyl-3-methylimidazolium tetrafluoroborate and liquid paraffin	LOD 9 nM and 0.5 μM in buffer (not reported in serum); linear DR of 0.07 to 500 μM, 9.0 nM to 0.5 μM in buffer; recoveries of 98.1 to 102.3%	[67]
Acetaminophen and etilefrine (with dopamine)	DPV; glassy carbon electrodes; oxidation of each of small molecule	Serum-10-fold dilution followed by another 125-fold dilution	Platinum-nickel nanoparticles and reduced graphene oxide	LOD 8.2 μM, 14.9 μM, 0.0025 μM in buffer (not reported in serum); linear DR of 4.0 to 60 μM, 4.0 to 100 μM, 0.05 to 0.5 μM in buffer; recoveries of 95 to 108%	[68]
Dobutamine and amlodipine for the treatment of cardiac issues and acetaminophen and ascorbic acid	DPV; glassy carbon electrodes; oxidation of each species	Serum-20-fold dilution	Composite of multi-walled carbon nanotubes, ionic liquid crystal, graphene and 18-Crown-6 enables the simultaneous detection of four species	LOD 0.50 nM, 0.14 nM, 0.09 nM, 9.2 nM; linear DR of 0.02 to 40 μM, 0.008 to 30 μM, 0.001 to 20 μM, 0.4 to 40 μM; recoveries of 97.1 to 102.7%	[62]
Acetaminophen (with tryptophan and caffeine)	DPV; glassy carbon electrodes; oxidation of each species	Serum-25-fold dilution	Tin sulfide and titanium dioxide on graphene oxide sheets	LOD 7.5 nM, 7.8 nM, 4.4 nM in buffer (not reported in serum); linear DR of 9.8 nM to 280 μM, 13 nM to 157 μM, 16 nM to 333 μM in buffer; recoveries of 98% and 99% for acetaminophen	[69]
Diclofenac sodium as an analgesic and anti-inflammatory for arthritis and other conditions	DPV and CV; glassy carbon electrodes; oxidation of diclofenac sodium	Serum-filtered and diluted 10-fold	Nanocellulose and multi-walled carbon nanotubes	LOD 0.12 μM in buffer (not reported in serum); linear DR of 0.05 to 1 μM in buffer; recoveries of 99.3 to 102.0%	[65]
Doxorubicin and dasatinib for the treatment of breast cancer	AdSSWV; glassy carbon electrodes; oxidation of each drug	Serum-10-fold dilution	Palladium and platinum nanoparticles with multi-walled carbon nanotubes	LOD 0.86 nM, 6.72 nM in buffer (not reported in serum); linear DR of 4.4 nM to 8.6 μM, 38 nM to 9.9 μM in buffer; recoveries of 99.1 to 100.6%	[70]
N-acetylcysteine for multiple indications	DPV; carbon paste electrodes; oxidation of each drug	Serum-10-fold or greater	Silica nanoparticles and boron trifluoride and 4,4’-dihydroxybiphenyl	LOD 0.33 μM in buffer (not reported in serum); linear DRs of 1.0 to 41.5 μM and 41.5 to 101.5 μM in buffer; agreed to within 1% of HPLC	[71]
Chloroquine to treat malaria, rheumatoid arthritis, and cancer	CV and DPV; glassy carbon electrodes; oxidation of chloroquine	Serum-5-fold dilution	Tungsten disulfide quantum dots with reduced graphene oxide	LOD 0.04 μM; linear DR of 0.5 to 82 μM	[72]
Olanzapine for the treatment of schizophrenia	Potentiometric measurement and carbon paste electrodes	Serum-10-fold dilution	Olanzapine-tungstophosphate	LOD 0.5 μM in buffer (not reported in serum); linear DR of 0.75 to 560 μM in buffer; recoveries of 97.8 to 101.6%	[39]
Epinephrine to treat allergic reactions, cardiac arrest, and hypertension	DPV; glassy carbon electrodes; oxidation of drugs	Serum-proteins separated out with ethanol precipitation and centrifugation	Zinc oxide nanoparticles and multi-walled carbon nanotubes	LOD 0.016 μM in buffer (not reported in serum); linear DR of 0.4 to 2.4 μM in buffer; recoveries of 100.4 to 101.3%	[41]
Azithromycin for the treatment of bacterial infections	DPV; glassy carbon electrodes; oxidation of azithromycin	Plasma-filtered using 0.45 μm filter and diluted 10-fold	Molecularly imprinted polymer	LOD 0.85 nM in buffer (not reported in serum); linear DR of 13 nM to 67 μM in buffer; recovery of 102.4%	[73]
Epirubicin and methotrexate for breast cancer treatment	DPV; glassy carbon electrodes; oxidation of each drug	Serum-filtered using 0.45 μm filter and diluted 5-fold	Zinc oxide nanoflowers doped with cerium	LOD 2.3 nM, 6.3 nM in buffer (not reported in serum); linear DR of 0.01 to 600 μM, 0.01 to 500 μM in buffer; recoveries of 98.0 to 102.3%	[74]
Rifampicin to treat bacterial infections	DPV; glassy carbon electrodes; oxidation of drugs	Serum-indicated dilution of 3-fold	Titanium dioxide nanoparticles on reduced graphene oxide	LOD 0.03 μM in buffer (not reported in serum); linear DR of 0.01 to 0.1 nM in buffer; recoveries of 95 to 100%	[75]
Levofloxacin for treating bacterial infections	Potentiometric measurement; carbon paste electrodes	Serum-diluted 25-fold	PVC coating	LOD 10 μM in buffer (not reported in serum); linear DR of 10^−2^ to 10^−4^ M in buffer; recoveries of 95.6 to 98.7% for CPE	[76]
Mefenamic acid, a non-steroidal anti-inflammatory drug	CV and DPV; carbon paste electrodes; oxidation of mefenamic acid	Serum-handling not described	Copper vanadium oxide nanostructures (Cu_5_V_2_O_10_)	LOD 2.3 nM in buffer (not reported in serum); linear DR of 0.01 to 470 μM in buffer; recoveries of 98.3 to 110%	[77]
Mefenamic acid, a non-steroidal anti-inflammatory drug	CV and DPV; carbon paste electrodes; oxidation of mefenamic acid	Serum-handling not described	Terbium titanate nanostructures (Tb_2_Ti_2_O_7_)	LOD 2.4 nM in buffer (not reported in serum); linear DR of 0.01 to 400 μM in buffer; recoveries of 92.0 to 107%	[78]

SWV, square-wave voltammetry; DPV, differential pulse voltammetry; CA, chronoamperometry; CV, cyclic voltammetry; AdSSWV, adsorptive stripping square-wave voltammetry; LOD, limit of detection; DR, dynamic range.

**Table 4.** Summary of advances in drug detection in alternative complex nonblood biological matrices.

Drug and Health Condition	Electrochemical Method/Base Working Electrode/Sensing Mechanism	Complex Biofluid	Strategies to Improve Electrochemical Signal	Performance Metrics (Note that Metrics are Provided for Each of the Targets in the Order Listed in Column 1)	Ref.
Interferon gamma for treating cancer and infections	Amperometry; screen-printed carbon electrodes treated with p-ABA diazonium salt to immobilize capture Ab; reduction of benzoquinone from an enzymatic reaction of label HRP, hydroquinone, and H_2_O_2_	Saliva collected with a Salivette and then extracted using centrifugation	Optimization of parameters including the capture Ab concentration and the concentrations of detected Ab and enzymes	LOD 1.6 pg/mL in buffer; linear DR of 2.5 to 2000 pg/mL in buffer; in saliva, measurements agreed with ELISA to within 3%	[97]
Carbamazepine to treat epilepsy seizures	SWV; stencil-printed carbon electrodes; carbamazepine oxidation	Saliva—pooled, commercially purchased	Sodium dodecyl sulfate in solution	LOD 1 μM; average QR of 0.85 μM from 0 to 15 μM	[79]
	SWV; stencil-printed carbon electrodes; carbamazepine oxidation	Saliva—pooled, commercially purchased	Sodium dodecyl sulfate film on electrodes	LOD 1 μM; average QR of 1.6 μM from 0 to 15 μM for field-use format sensor	[80]
Acetaminophen/paracetamol as an analgesic	DPV; oxygen-terminated boron-doped diamond electrode; oxidation of acetaminophen	Saliva and sweat—saliva was processed by centrifugation before use	Hydrogen-terminated boron-doped diamond electrode with a Nafion layer	LOD 1 μM; strong correlation in saliva (*R*^2^ = 0.92) and sweat (*R*^2^ = 0.95) with LC-MS/MS	[82]
Acetaminophen/paracetamol to manage pain	DPV; screen-printed carbon electrodes; oxidation of acetaminophen	Saliva, unprocessed	Electrochemical pretreatment consisting of cyclic voltammetry of 0.5 M sulfuric acid increased the electrode conductivity and signal	LOD 14.5 μM; linear DR 25 to 150 μM	[81]
Benzodiazepine for the treatment of depression, anxiety, and insomnia	DPV; laser-scribed graphene electrodes functionalized with Ab capture; oxidation current change with Ab-Ag binding	Saliva collected with a swab and extracted by centrifugation	Optimization of capture of the Ab concentration and blocking agent treatment	LOD 9.7 ng/mL in buffer; DR of 1.0 pg/mL to 500 ng/mL in buffer; simultaneous detection with amphetamine and cocaine in saliva	[98]
Levodopa for the treatment of Parkinson’s disease	Amperometry; gold-coated electrodes with tyrosinase; tyrosinase-catalyzed oxidation of levodopa	Simulated sweat using iontophoretic stimulation or exercise	Gold nanodendrite structures on gold electrodes and Nafion film	LOD 1 μM; DR of 0 to 20 μM; sensitivity of 17 nA/μM	[83]
	Amperometry; gold-coated electrodes with tyrosinase; tyrosinase-catalyzed oxidation of levodopa	Sweat generated at rest	Gold nanodendrite structures on the gold electrodes and the Nafion-TBAB film	LOD 3 μM in buffer; linear DR of 0 to 50 μM in buffer; in situ sweat analysis	[84]
Levodopa for treatment of Parkinson’s disease	Amperometry; screen-printed carbon electrodes coated with crosslinked tyrosinase; dopaquinone reduction	Fingertip sweat using a touch sensor	Note that an advantage to their method is its robustness to fouling of the electrode via unintended quinone polymerization reactions	LOD 300 nM in buffer; linear DR of 1 to 30 μM in buffer; in situ sweat analysis	[85]
Acetaminophen/paracetamol to manage pain and paroxetine as an antidepressant	DPV; screen-printed carbon electrodes	Artificial sweat and human sweat combined with artificial sweat in equal parts	Pretreatment consisting of cyclic voltammetry of 0.5 M sulfuric acid	LOD 0.25 μM, 0.49 μM in artificial sweat; recoveries of 106% and 112% in artificial sweat	[86]
Levodopa for treatment of Parkinson’s disease	SWV and CA; carbon paste electrodes with tyrosinase in microneedles; oxidation of levodopa	Artificial interstitial fluid	Nafion	LOD 0.5 μM in artificial ISF; linear DR of 0.5 to 3 μM in artificial ISF	[88]
Apomorphine for treatment of Parkinson’s disease	SWV and CA; carbon paste electrodes in microneedles; apomorphine oxidation	Artificial interstitial fluid containing protein interferents	2% rhodium nanoparticles and 1% Nafion film for stability against protein interferents	LOD 0.6 μM/0.75 μM (SWA/CA) in buffer; linear DR of 10 to 60 μM in the skin mimic model; sensitivity of 3.8 nA/μM in the skin mimic model	[89]
Tobramycin and vancomycin for bacterial infections	SWV; gold coating of an acupuncture needle; the binding of aptamer to the antibiotic reduces the distance between the methylene blue tag and the electrode and increases the current signal	Interstitial fluid—in vivo on rat	Gold nanoparticle coating enhances the signal compared to evaporated gold film	Response curves from 0 to 100 μM for each in artificial ISF; correlation between blood and ISF tobramycin levels in mice	[90]
Cyproterone acetate for the treatment of prostate cancer	SWV; glassy carbon paste electrodes; reduction of cyproterone acetate	Urine—diluted with buffer	Multi-walled carbon nanotubes and gold nanoparticles	LOD 17.9 nM; linear DR of 99 nM to 5.0 μM; sensitivity 173 μA/μM per cm^2^	[64]
Diclofenac sodium as an analgesic and anti-inflammatory for arthritis and other conditions	DPV; glassy carbon electrodes; oxidation of diclofenac sodium	Urine—diluted 4-fold	Nanocellulose and multi-walled carbon nanotubes	LOD 0.12 μM in buffer (not reported in urine); linear DR of 0.05 to 1 μM in buffer; recoveries of 98.0 to 104.0%	[65]
Doxorubicin and dasatinib for the treatment of breast cancer	AdSSWV; glassy carbon electrodes; oxidation of each drug	Urine—10-fold dilution	Palladium and platinum nanoparticles with multi-walled carbon nanotubes	LOD 0.86 nM, 6.72 nM in buffer (not reported in urine); linear DR of 4.4 nM to 8.6 μM, 38 nM to 9.9 μM in buffer; recoveries of 98.8 to 99.5%	[70]
Diclofenac sodium as an analgesic and anti-inflammatory for arthritis and other conditions	DPV; screen-printed carbon electrodes; oxidation of diclofenac sodium	Urine—centrifuged to remove solids	Platinum nanoflowers with reduced graphene oxide facilitated additional analyte on the electrode and improved electron transfer	LOD 40 nM in buffer (not reported in urine); linear DR 0.1 to 100 μM in buffer; recoveries of 84 to 105%	[92]
Azithromycin for the treatment of bacterial infections	DPV; glassy carbon electrodes; oxidation of azithromycin	Urine and tears—filtered using an 0.45 μm filter and diluted 10-fold	Molecularly imprinted polymer	LOD 0.85 nM in buffer (not reported in urine); linear DR of 13 nM to 67 μM in buffer; recoveries of 98.0 to 106.3%	[73]
Epirubicin and methotrexate for breast cancer treatment	DPV; glassy carbon electrodes; oxidation of each drug	Urine—filtered using 0.45 μm filter and diluted 5-fold	Zinc oxide nanoflowers doped with cerium	LOD 2.3 nM, 6.3 nM in buffer (not reported in urine); linear DR of 0.01 to 600 μM, 0.01 to 500 μM in buffer; recoveries of over 97.1 to 102.6%	[74]
Levofloxacin for treating bacterial infections	Potentiometric measurement; carbon paste electrodes	Urine—diluted 25-fold	PVC coating	LOD 10 μM in buffer (not reported in urine); linear DR of 10^−2^ to 10^−4^ M in buffer; recoveries of 94.5 to 98.4% for CPE	[76]
Triamterene as a diuretic	CV, CA, SWV; boron-doped diamond electrodes; reduction of triamterene	Pooled and individual urine	Note that the electrode choice provides resistance to biofouling, stability, and a relatively large potential window	LOD 7.80 nM pooled and 20.8 nM individual urine	[91]
Nalbuphine as an analgesic	Potentiometric measurement; screen-printed carbon electrodes	Urine—diluted 10-fold	Composite of polyaniline with multi-walled carbon nanotubes and PVC with molecularly imprinted polymer beads	LOD 11 μM in buffer (not reported in urine); linear DR of 43 to 3300 μM in buffer; recoveries of 91.0 to 101.5%	[94]
Methotrexate for cancer treatment	DPV; screen-printed graphite electrodes; oxidation of methotrexate and folic acid	Urine—centrifuged, supernatant filtered using 0.45 μm filter and diluted at least 2.5-fold	Composite of iron oxide and polypyrrole and palladium	LOD 7.0 nM in buffer (not reported in urine); linear DR of 0.03 to 100 μM in buffer; recoveries of 97.8 to 103.1%	[93]
Sulfanilamide for the treatment of bacterial infections	CV and DPV; 3D printed carbon black-PLA electrodes; oxidation of sulfanilamide	Artificial urine—diluted 10-fold	Electrode pretreatment of NaOH solution at 1.4 V and −1 V for 200 s each	LOD 12 nM in buffer; linear DR of 1 to 39 μM in buffer; recoveries of 99.1 to 101.9% in synthetic urine	[99]
Trimethoprim for the treatment of bacterial infections	DPV; carbon paste electrodes with iron oxide and multi-walled carbon nanotubes; oxidation of trimethoprim	Urine—centrifuged and supernatant analyzed	Layered structure consisting of base electrode material and reduced graphene oxide and molecularly imprinted polymer with iron oxide and multi-walled carbon nanotubes	LOD 1.2 nM in buffer (not reported in urine); linear DRs of 0.004 to 0.08 μM and 0.08 to 500 μM in buffer; recoveries of 95.0 to 110.0%	[95]
Aminophylline for the treatment of bronchial asthma	DPV; glassy carbon electrodes; oxidation of aminophylline	Urine—filtered and diluted	Molecularly imprinted polymer and graphene oxide	LOD 2.1 pM in buffer (not reported in urine); linear DR of 37 pM to 1 mM in buffer; recoveries of 98.2 to 99.6%	[96]
Ketoconazole for the treatment of fungal infections	DPV, CV, CA; carbon paste electrodes; oxidation of ketoconazole	Urine—centrifuged, filtered, and diluted	Metal-organic framework composed of cerium and 1,3,5 benzene tricarboxylic acid and ionic liquid	LOD 0.04 μM in buffer; linear DR of 0.1 to 110 μM in buffer; recoveries of 96.7 to 102.0%	[100]

SWV, square-wave voltammetry; DPV, differential pulse voltammetry; CA, chronoamperometry; CV, cyclic voltammetry; AdSSWV, adsorptive stripping square-wave voltammetry; LOD, limit of detection; QR, quantitative resolution; DR, dynamic range.
